# Endocytosis of Connexin 36 is Mediated by Interaction with Caveolin-1

**DOI:** 10.3390/ijms21155401

**Published:** 2020-07-29

**Authors:** Anna Kotova, Ksenia Timonina, Georg R. Zoidl

**Affiliations:** 1 Department of Biology, York University, Toronto, ON M3J 1P3, Canada; kotova@my.yorku.ca (A.K.); ktimonin@yorku.ca (K.T.); 2 Department of Psychology, York University, Toronto, ON M3J 1P3, Canada

**Keywords:** connexin 36, caveolin-1, lipid raft, protein interaction, transport, FRET, TIRF

## Abstract

The gap junctional protein connexin 36 (Cx36) has been co-purified with the lipid raft protein caveolin-1 (Cav-1). The relevance of an interaction between the two proteins is unknown. In this study, we explored the significance of Cav-1 interaction in the context of intracellular and membrane transport of Cx36. Coimmunoprecipitation assays and Förster resonance energy transfer analysis (FRET) were used to confirm the interaction between the two proteins in the Neuro 2a cell line. We found that the Cx36 and Cav-1 interaction was dependent on the intracellular calcium levels. By employing different microscopy techniques, we demonstrated that Cav-1 enhances the vesicular transport of Cx36. Pharmacological interventions coupled with cell surface biotinylation assays and FRET analysis revealed that Cav-1 regulates membrane localization of Cx36. Our data indicate that the interaction between Cx36 and Cav-1 plays a role in the internalization of Cx36 by a caveolin-dependent pathway.

## 1. Introduction

The intracellular transport of connexins, their assembly and channel formation, and removal are governed by complex interactions with regulatory, transport, and structural proteins [1,2] The turnover of connexins from the cell membrane is, in particular, challenging for connexin 36 (Cx36), the major component of electrical synapses. In these gap junctions of the brain, Cx36 has been found in axo-axonal, axo-dendritic, and dendro-dendritic contact sites [3,4,5]. It is reasonable to expect that each type of contact site presents a distinct local environment with both unique and shared complements of Cx36-interacting proteins enabling on-demand protein transport and removal. 

One of the most notable interacting partners of Cx36 is the Ca^2+^/calmodulin dependent protein kinase II (CaMKII) [6]. Cx36 also exhibits a unique property called the “run-up” phenomenon, in which its conduction increases 10-fold [7]. The deletion of CaMKII binding and phosphorylation regions in Cx36 led to the loss of this “run-up” property, signifying that this interaction is essential for the functional plasticity of electrical synapses formed by Cx36. Calmodulin (CaM), another multifunctional calcium signaling protein, has also been shown to bind Cx36 [8]. Both CaMKII and CaM share a binding motif and interact with Cx36 competitively. Cx36 also interacts with scaffolding proteins [9], proteins of the zonula occludens family [10,11], or protein kinases [12,13]. A recent study demonstrated that Cx36 interaction with tubulin potentiates the synaptic strength of Cx36 by tubulin-mediated delivery of channels to the gap junction plaques [14]. 

Several connexins, including Cx36, have been shown to interact with a membrane/lipid raft protein called Caveolin-1 (Cav-1) [15,16]. However, the functional role of the Cx36/Cav-1 interaction has yet to be established. Caveolins are the primary components of caveolae and are involved in cellular processes such as transcytosis, potocytosis, endocytosis, and signal transduction [17]. While caveolae, the flask-like invaginations of the plasma membrane, are known to exist in numerous cell types except for neurons, caveolins can be expressed in neurons independently of caveolae [18]. This family of proteins is composed of three members: caveolin-1, caveolin-2, and caveolin-3, which have been reported to be expressed in many cell types, including neurons [19,20]. Analogous to Cx36 [21], Cav-1 is expressed in hippocampal neurons [22]. Recent studies have linked the expression of caveolins in the brain to the regulation of various neuronal processes, including hippocampal plasticity [23,24]. Lipid rafts are vital for synapse development, maintenance, and stabilization [25,26]. Cav-1 targets various neurotrophic receptors such as NMDA, AMPA, Trk, and GPC, [27,28,29,30] to the rafts, and also regulates components of the actin cytoskeleton [28]. Cav-1 has also been shown to regulate the activity of several channels [31,32,33], and it has been suggested that the rafts might be involved in connexin trafficking [34]. 

Here, the role of Cav-1 in regulating the Cx36 function was investigated in the Neuro 2a cell line. We demonstrated that the Cx36/Cav-1 interaction is calcium-dependent using Förster Resonance Energy Transfer Analysis (FRET) and coimmunoprecipitation (CoIP). Total Internal Reflection Fluorescence (TIRF) and Fluorescence Recovery After Photobleaching (FRAP) determined the role of Cav-1 on intracellular transport and membrane dynamics of Cx36. Pharmacological interventions, together with FRET and cell surface biotinylation assays confirmed the involvement of Cav-1 in the endocytosis of Cx36. Our results suggest that an increased Cx36/Cav-1 interaction may be a key mechanism implicated in the caveolin-dependent endocytosis of Cx36. We expect that these findings have implications for the spatial regulation of Cx36 and its turnover in the plasma membrane.

## 2. Results

### 2.1. Cx36 Co-Localizes With and Is in Close Proximity to Cav-1 in Neuro 2a Cells

**Figure 1:**
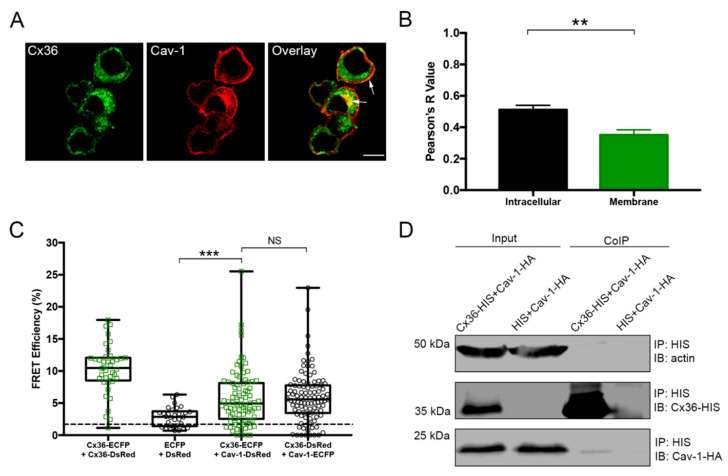
Co-localization, Förster Resonance Energy Transfer Analysis (FRET), and coimmunoprecipitation (CoIP) analysis of Cx36 and Cav-1. (**A**) Neuro 2a cells transfected with Cx36-HIS and Cav-1-HA were labeled with anti-HIS and anti-HA antibodies. Cx36 and Cav-1 displayed co-localization in the intracellular compartments and partly at the membrane (white arrows). Scale bar: 10 µm. (**B**) Co-localization quantification of the Cx36-HIS and Cav-1-HA intracellularly and at the membrane. Error bars show standard error of the mean. Sample sizes were the following: Intracellular: *n* = 27; Membrane: *n* = 27. (**C**) FRET efficiencies. Cx36-ECFP and Cx36-DsRed pair served as positive control while ECFP and DsRed pair served as a negative control. Cx36-ECFP and Cav-1-DsRed pair showed high FRET efficiency, signifying that two proteins are close to one another. The exchange of tags on both proteins (Cx36-DsRed and Cav-1-ECFP) had no significant effect on FRET efficiency. The dotted line represents the threshold of 1.7% (equals to 10 nm distance between FRET pairs). Error bars show the minimum and maximum values. Sample sizes were the following: Cx36-ECFP + Cx36-DsRed: *n* = 40; ECFP + DsRed: *n* = 30; Cx36-ECFP + Cav-1-DsRed: *n* = 86; Cx36-DsRed + Cav-1-ECFP: *n* = 100. (**D**) CoIP of Cx36 and Cav-1. HIS antibody was used to pull down the Cx36-HIS and Cav-1-HA complex. Neuro 2a cells double transfected with HIS and Cav-1-HA served as a negative control. Input lanes (cell lysates) show protein levels prior to the assay. CoIP lanes represent eluted protein complexes. A low amount of Cav-1 eluted together with Cx36 signified weak or transient interaction. Anti-HA and anti-HIS antibodies detected Cav-1 and Cx36 proteins, respectively. An anti-ß-actin antibody served as a loading control. IP: immunoprecipitation; IB: immunoblotting. ** *p* < 0.01, *** *p* < 0.001, NS—not significant.

To investigate the co-localization patterns of Cx36 and Cav-1, Neuro 2a cells were double transfected with Cx36-HIS and Cav-1-HA. Proteins were labeled with the corresponding primary antibodies and imaged 48 h post-transfection. Cx36 and Cav-1 co-localize in the intracellular compartments and partly at the cell membrane (Figure 1A, arrows). Co-localization quantification revealed that Cx36 and Cav-1 co-localize significantly more intracellularly than at the membrane (Intracellular: 0.51 ± 0.028, *n* = 27; Membrane: 0.35 ± 0.032, *n* = 27; *p* = 0.0010) (Figure 1B).

Neuro 2a cells were double transfected with Cx36-ECFP and Cav-1-DsRed to investigate proximity between these two proteins using FRET. FRET efficiency above the threshold of 1.7% signified that proximity between two proteins is less than 10 nm, meaning that they are close enough to interact with each other (Figure 1C). Because Cx36 monomers oligomerize into hexamers, Cx36-Cx36 pairs served as a positive control with a FRET efficiency value of 10.27 ± 0.60 (*n* = 40). Cells transfected with fluorescent tags alone served as a negative control. ECFP-DsRed pair displayed the FRET efficiency value of 2.82 ± 0.28 (*n* = 30). The value above the threshold can be explained by the partial dimerization of the fluorescent tags. FRET efficiency between Cx36-ECFP and Cav-1-DsRed pair was 5.73 ± 0.47 (*n* = 86) and was significantly different from the negative control group (*p* = 0.0001). This result proved that the two proteins were close enough to interact with each other. To assess whether the protein tags have an equal impact on the interaction, the tags were switched (Cx36-DsRed and Cav-1-ECFP). FRET efficiency was not significantly different (5.94 ± 0.40, *n* = 100, *p* = 0.4538), suggesting that protein tags are interchangeable and have minimal impact on FRET efficiency. 

To confirm the interaction between Cx36 and Cav-1, a CoIP assay was performed. Neuro 2a cells were double transfected with Cx36-HIS and Cav-1-HA, and the HIS antibody was used to pull down the protein complexes. The expression of the proteins of interest in the lysate (input) and elution (CoIP) fractions was confirmed with western blot analysis (Figure 1D). Low levels of Cx36 coimmunoprecipitated with Cav-1, suggesting a weak or transient interaction. 

### 2.2. Calcium Enhances the Interaction Between Cx36 and Cav-1

**Figure 2:**
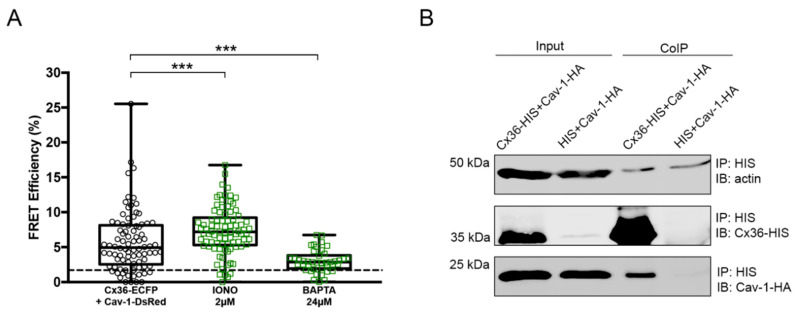
Interaction between Cx36 and Cav-1 is strengthened upon Ionomycin incubation. (**A**) FRET efficiencies of the Cx36-ECFP and Cav-1-DsRed pair under the action of Iono and BAPTA pharmacological agents. The dotted line represents the threshold of 1.7% (equals to 10 nm). Error bars show the minimum and maximum values. Sample sizes were the following: Cx36-ECFP + Cav-1-DsRed: *n* = 86; Iono: *n* = 85; BAPTA: *n* = 42. (**B**) CoIP of Cx36-HIS and Cav-1-HA after stimulation with Iono. HIS antibody was used to pull down the Cx36-HIS and Cav-1-HA complex. Neuro 2a cells double transfected with HIS and Cav-1-HA served as a negative control. Input lanes (cell lysates) show protein levels prior to the assay. CoIP lanes represent eluted protein complexes. Cav-1 eluted together with Cx36, signifying that calcium is required to strengthen the interaction between the two proteins. Anti-HA and anti-HIS antibodies detected Cav-1 and Cx36 proteins, respectively. An anti-ß-actin antibody served as a loading control. IP: immunoprecipitation; IB: immunoblotting. ***p < 0.001.

Due to the existing relationship between calcium and Cx36, we tested whether an influx of intracellular calcium would strengthen the interaction between Cx36 and Cav-1. We first tested the effect of Ionomycin (Iono) and 1,2-bis (2-aminophenoxy) ethane-N, N, N’, N’-tetraacetate (BAPTA) pharmacological agents on FRET efficiency between Cx36 and Cav-1 (Figure 2A). Treatment with 2 μM Iono, a calcium ionophore, significantly increased FRET efficiency between Cx36-ECFP and Cav-1-DsRed pairs (7.33 ± 0.37, *n* = 85, *p* = 0.0004). Treatment with 24 μM BAPTA, a calcium chelator, served as a negative control to Iono and significantly decreased FRET efficiency (3.015 ± 0.25, *n* = 42, *p* = 0.0001).

Because Iono enhanced the FRET efficiency between Cx36 and Cav-1 and BAPTA showed the opposite effect, the involvement of calcium in Cx36 and Cav-1 interaction was further explored. The possibility that calcium would strengthen the interaction between these two proteins was tested using CoIP assay. Prior to lysing, cells were treated with 2 μM Iono for 10 min. HIS antibody was used to pull down the protein complexes. Both Cx36 and Cav-1 were found in the elution fraction (Figure 2B). This assay further confirmed that an increase in the intracellular calcium strengthens the interaction between Cx36 and Cav-1. 

### 2.3. Cx36 and Cav-1 Co-Localize More With Golgi Than the ER and Their Interaction is Reduced With BFA Treatment

**Figure 3:**
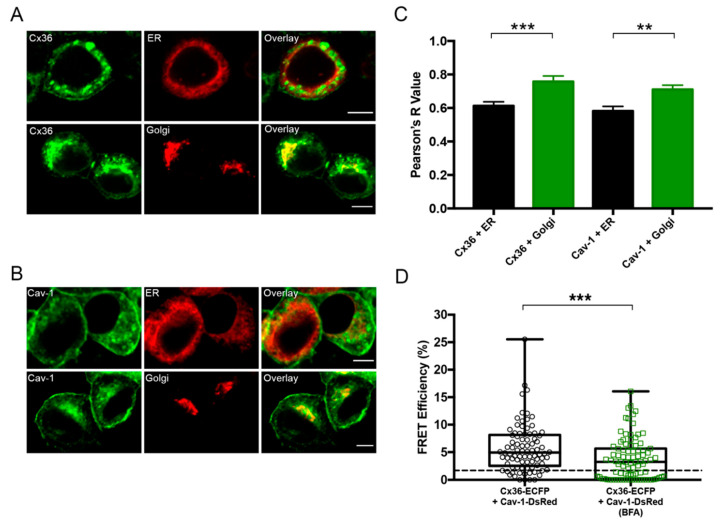
Co-localization with cellular markers and FRET analysis of Cx36 and Cav-1 post Brefeldin A (BFA) treatment. (**A**,**B**) Co-localization of Cx36-HIS and Cav-1-HA with DsRed-tagged calreticulin (ER marker), and DsRed-tagged galactosyltransferases (Golgi apparatus marker). HIS-tagged Cx36 was detected using an anti-HIS antibody and HA-tagged Cav-1 was detected using an anti-HA antibody. Alexa Fluor 568 was used as a secondary antibody. Scale bar: 5 µm (**C**). Co-localization quantification of the Cx36 and Cav-1 with the organelle markers. Error bars show standard error of the mean. Sample sizes were the following: Cx36 and ER: *n* = 30; Cx36 and Golgi: *n* = 20; Cav-1 and ER: *n* = 21; Cav-1 and Golgi: *n* = 19. (**D**) FRET efficiencies of Neuro 2a cells transfected with Cx36 and Cav-1 with and without BFA treatment. The threshold of 1.7% (equals to 10 nm) is represented by the dotted line. Error bars show the minimum and maximum values. Sample sizes were the following: Cx36-ECFP + Cav-1-DsRed: *n* = 86; BFA: *n* = 91. **p < 0.01, ***p < 0.001.

The next step was to determine in which intracellular location the Cx36/Cav-1 interaction occurs. Co-localization studies with the Golgi marker, galactosyltransferases, and the endoplasmic reticulum (ER) organelle marker, calreticulin, were performed. Neuro 2a cells were double transfected with the following pairs: Cx36-HIS and ER-DsRed, Cx36-HIS and Golgi-DsRed (Figure 3A), Cav-1-HIS and ER-DsRed and Cav-1-HIS and Golgi-DsRed (Figure 3B). Cx36 showed more co-localization with the Golgi marker than with ER (Cx36 and ER: 0.61 ± 0.024, *n* = 30; Cx36 and Golgi: 0.76 ± 0.034, *n* = 21; *p* = 0.0008) (Figure 3C). Cav-1 showed the same co-localization pattern (Cav-1 and ER: 0.58 ± 0.028, *n* = 21; Cav-1 and Golgi: 0.71 ± 0.024, *n* =19; *p* = 0.0018).

This result led us to believe that Cx36 and Cav-1 are likely to interact in the Golgi apparatus. To explore this idea, Brefeldin A (BFA), a pharmacological agent that blocks transport between ER and Golgi [35], was employed. Neuro 2a cells were transfected with Cx36-ECFP and Cav-1-DsRed and incubated with BFA for 6 h prior to FRET analysis. FRET efficiency between the Cx36 and Cav-1 post BFA treatment (3.76 ± 0.40, *n* = 91) was significantly decreased when compared to untreated cells (5.73 ± 0.47, *n* = 86, *p* = 0.0003) (Figure 3D). This result further confirmed that a population of Cx36 and Cav-1 proteins were interacting in the Golgi apparatus. 

### 2.4. Cav-1 Affects Both Vesicular and Membrane Transport of Cx36

**Figure 4:**
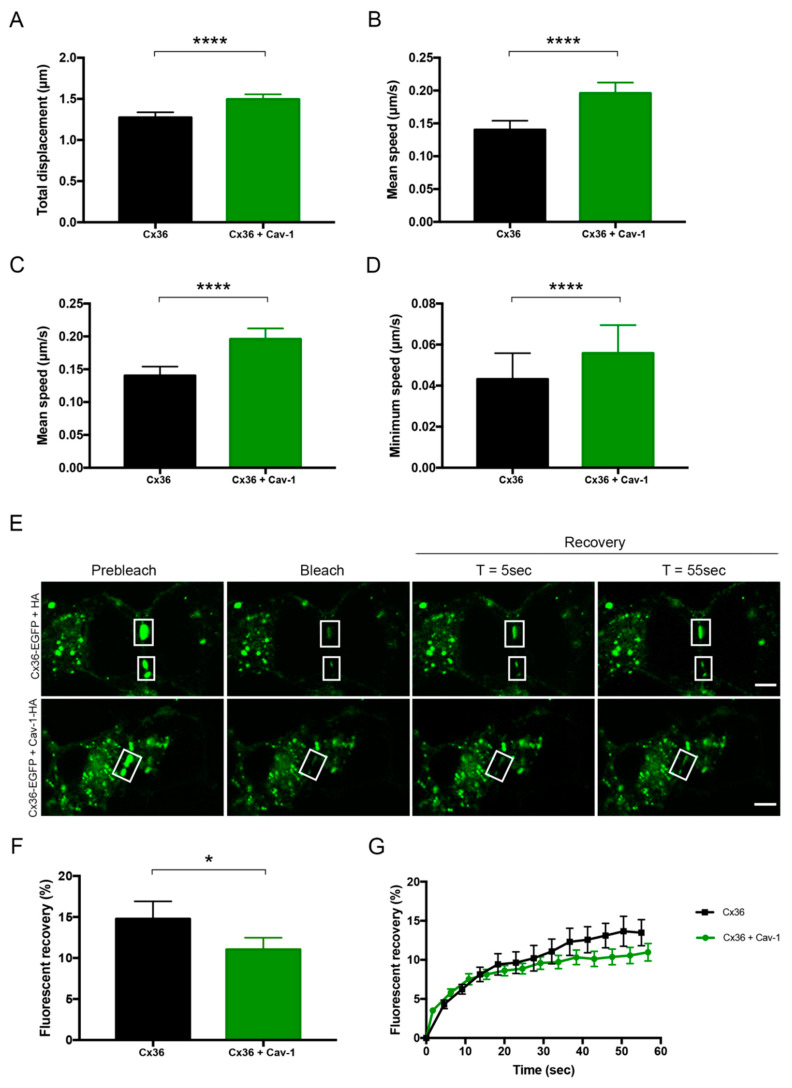
The effect of Cav-1 on vesicular transport and membrane dynamics of Cx36. Total Internal Reflection Fluorescence (TIRF) microscopy was used to resolve the vesicular transport of Cx36-EGFP. Cav-1 significantly amplified displacement (**A**) and the mean (**B**), maximum (**C**), and minimum speed of Cx36 (**D**). Error bars show standard error of the mean. Sample sizes were the following: Cx36-EGFP and HA: *n* = 1107; Cx36-EGFP and Cav-1-HA: *n* = 1349. (**E**) FRAP analysis of cells transfected with Cx36-EGFP and HA or Cx36-EGFP and Cav-1-HA showing selected regions (white rectangles) pre bleaching, immediately after bleaching, and recovery 5 and 55 s post bleaching. Scale bar: 5 µm. (**F**) The bar graph displays the total % fluorescent recovery of the selected gap junction regions, 55 s post bleaching. Error bars show standard error of the mean. Sample sizes were the following: Cx36-EGFP: *n* = 23; Cx36-EGFP and Cav-1-HA: *n* = 24. (**G**) Overall trends in % recovery measured every 5 s, over the 55-s duration. Error bars show standard error of the mean. *p < 0.05, ****p < 0.0001.

To further explore the importance of the interaction between Cx36 and Cav-1 we examined the effect of Cav-1 overexpression on the intracellular transport of Cx36. Neuro 2a cells transfected with Cx36-EGFP and Cav-1-HA or with Cx36-EGFP and HA were subjected to TIRF microscopy. Trafficking dynamics of the individual vesicles, illuminated in the submembrane space, were recorded over the 1-min duration (Figure 4A–D). Vesicles double transfected with both Cx36 and Cav-1 demonstrated increased displacement (Cx36: 1.28 ± 0.062, *n* = 1107; Cx36 and Cav-1: 1.50 ± 0.061, *n* = 1349; *p* < 0.0001) (Figure 4A), mean speed (Cx36: 0.14 ± 0.014, *n* = 1107; Cx36 and Cav-1: 0.20 ± 0.016, *n* = 1349; *p* < 0.0001) (Figure 1B), maximum speed (Cx36: 0.64 ± 0.037, *n* = 1107; Cx36 and Cav-1: 0.75 ± 0.037, *n* = 1349; *p* < 0.0001) (Figure 1C) and minimum speed (Cx36: 0.043 ± 0.013, *n* = 1107; Cx36 and Cav-1: 0.056 ± 0.014, *n* = 1349; *p* < 0.0001 (Figure 1D). 

To assess whether Cav-1 has an effect on the membrane dynamics and gap-junction regeneration of Cx36, FRAP microscopy was employed. Gap junctions were used as regions of interest (Figure 4E). FRAP analysis revealed that fluorescent recovery of the Cx36-EGFP and Cav-1-HA transfected cells was significantly lower than of Cx36-EGFP and HA transfected cells (Cx36: 14.77 ± 2.136, *n* = 27; Cx36 and Cav-1: 11.04 ± 1.444, *n* = 29; *p* = 0.0467) (Figure 4F). However, both types of cells exhibited the same trend in recovery (Figure 4G). These results demonstrated that while Cav-1 increases the dynamics of the intracellular transport of Cx36, it reduces the gap junction plaque recovery, signifying that a lower amount of Cx36 is reaching the membrane in the presence of Cav-1. Taken together, these results suggest that Cav-1 enhances the retrograde transport of Cx36.

### 2.5. Cav-1 Depletes Levels of Cx36 From the Membrane via Endocytosis

**Figure 5:**
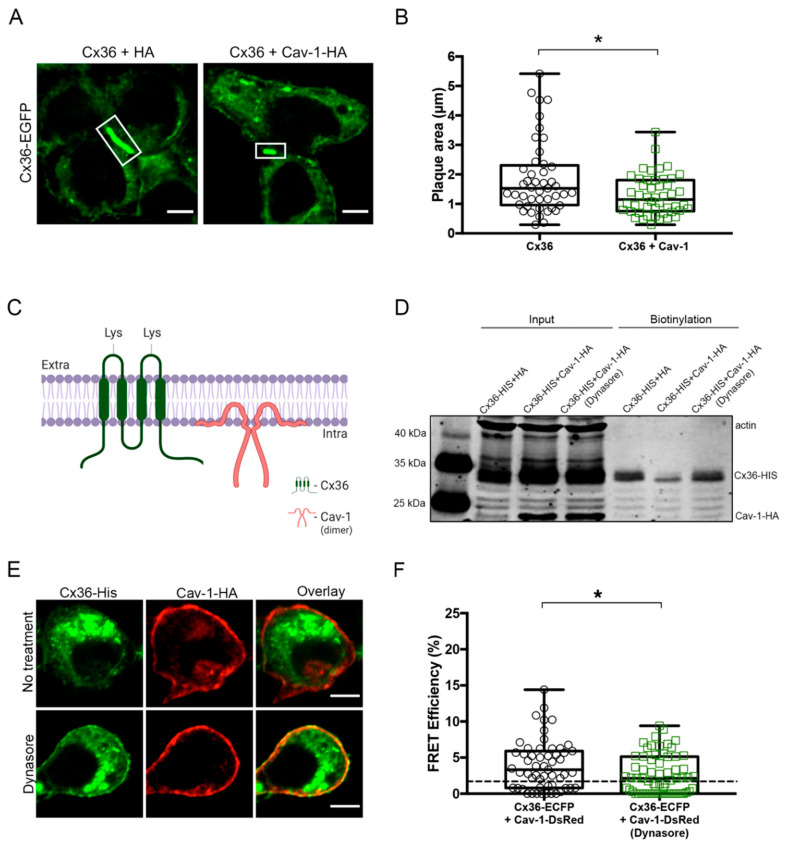
Cav-1 regulates levels of Cx36 at the membrane. (**A**) Representative images of Cx36 gap junction plaques (in white boxes) in live cells transfected with Cx36-EGFP and HA or Cx36-EGFP and Cav-1-HA. Scale bar: 5µm. (**B**) Gap junction plaque areas of cells double transfected with Cx36-EGFP and Cav-1-HA were significantly reduced when compared to cells transfected with Cx36-EGFP and HA. Error bars show the minimum and maximum values. Sample sizes were the following: Cx36-EGFP: *n* = 45; Cx36-EGFP and Cav-1-HA: *n* = 48. (**C**) Topological representation of Cx36 and Cav-1 structures. Unlike Cav-1, Cx36 possesses two extracellular loops which can undergo cell surface biotinylation. The image was created with BioRender.com. (**D**) Cell surface biotinylation assay displaying the effect of Cav-1 on levels of Cx36 at the membrane. Total cell lysates (Input) show expression of both Cx36 and Cav-1. The streptavidin pull-down fractions (Biotinylation) show that membrane levels of Cx36 were depleted in cells double transfected with Cav-1. Treatment with Dynasore restored membrane levels of Cx36 to baseline. Anti-HIS and anti-HA antibodies were used to detect Cx36 and Cav-1 proteins, and an anti-ß-actin antibody was used as an internal control. (**E**) Cx36 and Cav-1 transfected cells pre and post Dynasore treatment. Increased membrane expression of Cx36 can be observed post treatment. Scale bar: 5 µm. (**F**) FRET efficiencies of Neuro 2a cells transfected with Cx36 and Cav-1 with and without Dynasore treatment. The threshold of 1.7% (equals to 10 nm) is represented by the dotted line. Error bars show the minimum and maximum values. Sample sizes were the following: Cx36-ECFP + Cav-1-DsRed: *n* = 55; Dynasore: *n* = 64. *p < 0.05.

After establishing that Cav-1 has an effect on the intracellular transport of Cx36, we tested whether the same held for the membrane expression of Cx36. To assess the effect of Cav-1 on gap junction assembly or disassembly, the gap junction plaque area was measured in Cx36-EGFP and HA or Cx36-EGFP and Cav-1-HA transfected cells (Figure 5A). The gap junction plaque area of Cx36 and Cav-1 transfected cells was significantly lower when compared to Cx36 transfected cells (Cx36: 1.87 ± 0.19, *n* = 45; Cx36 and Cav-1: 1.28 ± 0.098, *n* = 48; *p* = 0.0230) (Figure 5B). 

To further compare levels of Cx36 at the membrane, cell surface biotinylation assay was performed. While Cx36 is a transmembrane protein containing two extracellular loops, Cav-1 does not contain extracellular domains; therefore, Cav-1 is unable to undergo cell surface biotinylation (Figure 5C). Cx36-HIS and HA or Cx36-HIS and Cav-1-HA transfected cells were biotinylated at the cell surface and pulled down with streptavidin (Figure 5D). Levels of Cx36 were notably lower when cells were also transfected with Cav-1, suggesting that Cav-1 depletes Cx36 from the membrane. To test whether this effect is due to the Cav-1 mediated endocytosis of Cx36, we employed the pharmacological agent Dynasore. Prior to cell surface biotinylation, cells transfected with Cx36 and Cav-1 were incubated with 50 μM Dynasore for 1 h. As expected, once endocytosis was blocked with Dynasore [36], levels of Cx36 were restored to baseline. Figure 5E displays the expected increase in the membrane expression of Cx36 once Dynasore is applied. Unlike Cx36, Cav-1 is predominately expressed in the membrane, therefore, a minor increase in the membrane localization is observed.

To confirm the specificity of this drug on the interaction, we examined the effect of Dynasore on FRET efficiency between Cx36 and Cav-1 (Figure 5F). Prior to the FRET analysis, cells transfected with Cx36-ECFP and Cav-1-DsRed were incubated with 50 μM Dynasore for 1 h. FRET efficiency between Cx36 and Cav-1 was significantly reduced upon Dynasore application, further suggesting that Cx36 and Cav-1 are interacting during the internalization pathway (no treatment: 4.00 ± 0.46, *n* = 55; Dynasore: 2.67 ± 0.33, *n* = 64, *p* = 0.0350). 

## 3. Discussion

Connexins have a short half-life of only a few hours [12,37,38] suggesting that efficient mechanisms must exist to control and facilitate on-demand genesis and removal from gap junctions. A previous study identified the lipid raft protein Cav-1 [15] as a candidate involved in the dynamic turnover of several connexins, including Cx36. Here, we employed Neuro 2a cells to characterize the interaction between Cx36 and Cav-1 further. CoIP and FRET analysis showed the interaction between both proteins. Various microscopy techniques, coupled with pharmacological interference, determined the role of Cav-1 in mediating endocytosis of Cx36. 

Connexins undergo internalization through clathrin-mediated endocytosis [39,40,41]. Entire or partial gap junction plaques are internalized as double-membrane vesicles, termed annular gap junctions or connexosomes [42,43]. Connexins have also been shown to localize within lipid rafts [15], suggesting the possibility of internalization through caveolae-dependent endocytosis. Gap junctions are usually much larger than lipid rafts [2], and the internalization of entire plaques by this alternative pathway is unlikely. Instead, under normal physiological conditions, connexins destined for the degradation are removed from the center of the plaque [44,45]. Endocytosis of Cx36 by a caveolin-mediated pathway might be one of the different pathways used by cells for dynamic control of gap junction mediated communication. 

Caveolins have been implicated in the internalization of several different proteins [46,47,48,49,50,51]. Re-expression of Cav-1 in Cav-1 negative cells resulted in increased endocytosis of β1 integrins and fibronectin [46]. We observed the same effect on Cx36, as Cav-1 overexpression resulted in an increased membrane depletion. Dynasore has been used effectively to inhibit caveolar endocytosis and prevented occludin internalization [47]. Here, Dynasore, an endocytosis inhibitor, counteracted the action of Cav-1. 

Some proteins, including glutamate transporters, rely on Cav-1 for both endocytosis and exocytosis [52]. The disruption of the Cav-1 function has been shown to cause intracellular retention and accumulation of glycosylphosphatidylinositol-linked proteins, angiotensin II type 1 receptor, and dysferlin [53,54,55]. Dysferlin is endocytosed rapidly in cells lacking Cav-1, signifying that Cav-1 is required for dysferlin’s retention at the cell surface [49]. In the case of Cx36, Cav-1 is not required for the trafficking to the cell surface, suggesting that alternative mechanisms facilitate exocytic transport. The interaction of Cx36 with tubulin is an example of this process [14].

Caveolin-1 has also been suggested to function as negative regulators of caveolae-dependent endocytosis [49,56,57]. However, this seems to be the mechanism for cell lines expressing stable levels of Cav-1. Cell lines with limited Cav-1 expression tend to show the opposite effect. In 293T cells, Cav-1 expression is below the detectable levels, and Cav-1 overexpression leads to an increased turnover of the TGF-β receptor [58]. Like 293T, Neuro 2a cells do not express detectable levels of Cav-1 [59,60]. Our results also indicate increased endocytosis with Cav-1 overexpression. This evidence suggests that Cav-1 is a positive regulator of Cx36 endocytosis in Neuro 2a cells. 

Both Cx36 and Cav-1 co-localized more efficiently with the Golgi apparatus than with ER. Further, the interaction between the two proteins was inhibited with BFA. BFA is widely used as an inhibitor of transport between ER and Golgi as it leads to Golgi disassembly [35]. However, BFA has also been shown to block the transport function of COPI vesicles [61], which are known to be involved in the retrograde recycling transport from Golgi to ER [62]. Analogous to the Golgi-ER fusion, the trans-Golgi network (TGN) fuses with the endosomal recycling system upon BFA addition [63]. TGN-endosome fusion impairs trafficking from endosomes, and the vesicles are retained in this compartment. FRET efficiency reduction between Cx36 and Cav-1 upon BFA incubation indicated that the proteins are retained in endosomes and are unable to interact in the Golgi apparatus during the retrograde pathway. The interaction was not fully abolished, as Cx36 and Cav-1 are likely interacting in the retained compartment or are interacting at the sites unaffected by BFA, such as vesicles leaving the membrane.

We also determined that the interaction between Cx36 and Cav-1 is calcium-dependent. Ionomycin raises intracellular calcium levels [64], and such conditions are typically found during synaptic activity in neurons [65]. Specifically for Cx36, Ionomycin significantly increases the extent of the “run-up,” and thus increases its conductance [7]. Interestingly, CaM is able to interact with Cx36 only when intracellular calcium levels are elevated [66]. A similar mechanism is indicative of Cx36 interaction with Cav-1, as the interaction levels increase with a rise in intracellular calcium. An influx of calcium has been shown to regulate intracellular transport. Also, the speed of both endocytosis and exocytosis is tightly controlled by intracellular calcium levels [67]. Specifically, in retinal bipolar cells, where Cx36 is highly expressed [68], calcium influx selects the fast mode of endocytosis at the synaptic terminals [69]. On the contrary, the application of BAPTA leads to membrane retrieval by a slower mechanism. Dynamin and synaptophysin, critical regulators of endocytosis, have been shown to interact in the presence of high concentrations of calcium [70]. Their interaction is indicative of a rapid and specialized mechanism of endocytosis. This is consistent with our results and supports that Cav-1 is a mediator of rapid endocytosis of Cx36.

The principal findings of this research provide insights into the life cycle of Cx36, specifically the regulation of its trafficking mechanisms. They highlight the role of Cav-1 in rapid, clathrin-independent endocytosis of Cx36 by maintaining the pool of releasable vesicles contributing to the dynamic functions of this connexin.

## 4. Materials and Methods

### 4.1. Plasmid Construction and Site-Directed Mutagenesis

The full-length *Rattus norvegicus* Cx36 [NM_019281, amino acids (aa) 1–321], isoform 2 of Cav-1 [NM_031969, (aa) 1–147], were cloned into pEGFP- N1, pECFP-N1, pDsRed-monomer, HA-N1 and HIS-N1 expression vectors (Clontech Laboratories Inc., Mountain View, CA, USA). Organelle markers for ER and Golgi apparatus were tagged with DsRed2 and generated as previously described [66]. All plasmid constructs used in this study were sequence verified (Eurofins, MWG Operon LLC, Huntsville, AL, USA)

### 4.2. Cell Culture and Transient Transfection

Mouse neuroblastoma 2a (Neuro 2a) cells (ATCC^®^, CCL-131, Manassas, VA, USA) were cultivated in Dulbecco’s Modified Eagle Medium (DMEM) supplemented with 10% fetal bovine serum (FBS), 1% penicillin and streptomycin, and 1% non-essential amino acids (all Thermo Fisher Scientific, Rockford, IL, USA) at 37 °C in a humidified atmosphere with 5% CO2. For coimmunoprecipitation assay, ~3,000,000 cells were seeded in one 100 mM plate. For the cell surface biotinylation assay, ~800,000 cells were seeded in 60 mM plates. For the live and fixed cell imaging, including FRET, ~25,000 cells were seeded in 35 mM glass-bottom dishes (MatTek Corporation, Ashland, MA, USA) or 24-well plates. Half an hour prior to the live microscopy assays, cells were transferred into DMEM lacking phenyl red.

Neuro 2a cells were transiently transfected with Effectene™ Transfection Reagent Kit (Qiagen Inc., Valencia, CA, USA) according to the manufacturer’s guidelines. Cells were double transfected with a total of 4000 ng or 1200 ng of DNA for each 100 mM or 60 mM plate, respectively. For 35 mM glass-bottom dishes and 24-well plates, cells were transfected with 200 ng for single transfections and 400 ng for double transfections. All of the experiments were performed 48 h post-transfection.

### 4.3. Pharmacology

Prior to imaging transfected cells were treated with the 2 μM Ionomycin (Sigma-Aldrich Chemie GmbH, Munich, Germany) and 24 μM of Ca2+ chelator BAPTA-AM (Thermo Fisher Scientific, Rockford, IL, USA), for 10 min. Cells were incubated with BFA (Sigma-Aldrich Chemie GmbH, Munich, Germany) at the concentration of 5 μg/mL for 6 h. Dynasore (Sigma-Aldrich Chemie GmbH, Munich, Germany) was used as an endocytosis inhibitor for 1 h at 50 μM concentration.

### 4.4. Western Blot

For western blot, cell protein lysates were prepared 48 h after transfection. Proteins were separated with 10% sodium dodecyl sulfate polyacrylamide gel electrophoresis (SDS-PAGE) at 150 V for 1.5 h. The gel was transferred to a nitrocellulose membrane using the Trans-Blot Turbo Transfer System (Bio-Rad Inc., Mississauga, ON, Canada) at 1.3 A and 2.5 V for 7 min. The membrane was washed in PBS buffer and blocked with Odyssey Blocking Buffer (LI-COR Biosciences, Lincoln, NE, USA) for 1 h at room temperature (RT). The membrane was then incubated with the primary antibody solution overnight at 4 ºC. The following primary antibodies were used for the western blot: rabbit anti-HIS (Bethyl Laboratories Inc., Montgomery, TX, USA) at 1:1000, mouse anti-HA (Roche Holding AG, Basel, Switzerland) at 1:500, mouse anti-β-actin (Sigma-Aldrich Chemie GmbH, Munich, Germany) at 1:1500 concentrations. The secondary antibodies, anti-mouse iRDye 800 and anti-rabbit iRDye 680 (LI-COR Biosciences, Lincoln, NE, USA), were used at 1:15,000 concentration. Imaging was performed using the Odyssey^®^ CLx Infrared Imaging System (LI-COR Biosciences, Lincoln, NE, USA).

### 4.5. Coimmunoprecipitation (CoIP)

Neuro 2a cells were double transfected with either Cx36-HIS and Cav-1-HA or HIS and Cav-1-HA. Transfected cells were lysed in IP Lysis buffer (Thermo Fisher Scientific, Rockford, IL, USA) supplemented with protease inhibitor cocktail kit (Thermo Fisher Scientific, Rockford, IL, USA). Lysates were centrifuged at 20,000× *g* for 10 min at 4 °C to remove the cell pellet. Cell lysates were precleared for 1 h at 4 °C with protein A-Sepharose (GE Healthcare, Chicago, IL, USA). The lysate was then transferred to a fresh tube and incubated with 10μg of anti-HIS antibody overnight at 4 °C. Next day lysate was combined with 100 μL of a 1:1 slurry of protein A-Sepharose beads and PBS containing 2% bovine serum albumin (BSA). Following a 2-h incubation at 4 °C, the mixture was centrifuged, washed two times with 1 mL of IP lysis buffer and three times with 1 mL of PBS. The proteins were eluted in a 1× Laemmli sample buffer for 5 min at 95 °C and subjected to western blot analysis.

### 4.6. Cell Surface Biotinylation Assay

Neuro 2a cells were seeded on 60 mM plates and transfected with Cx36-HIS and Cav-1-HA or Cx36-HIS and HA. Biotinylation assay was performed 48 h post-transfection. Cells were washed once with PBS containing both calcium and magnesium and labeled with 0.3 mg of membrane-impermeable EZ-linkTM Sulfo-NHS-Biotin (Thermo Fisher Scientific, Rockford, IL, USA) per plate for 30 min at room temperature. Plates were washed three times, 5 min each, with 50 mM glycine buffer to quench the reaction. Cells were then washed with PBS lacking calcium and magnesium and lysed with IP Lysis buffer (Thermo Fisher Scientific, Rockford, IL, USA) supplemented with protease inhibitor cocktail kit (Thermo Fisher Scientific, Rockford, IL, USA). Cell lysates were incubated overnight with 90 uL of DynabeadsTm MyONETm Streptavidin C1 (Invitrogen, Carlsbad, CA, USA) on a shaker at 4 ºC. The next day beads were collected on a magnet and washed using the following buffers: twice with buffer 1 (2% SDS in dH20), once with buffer 2 (0.1% deoxycholate, 1% Triton X-100, 500 mM NaCl, 1 mM EDTA, 50 mM Hepes pH 7.5), once with buffer 3 (250 mM LiCl, 0.5% NP-40, 0.5% deoxycholate, 1 mM EDTA, 10 mM Tris; pH 8.1), and twice with buffer 4 (50 mM Tris, 50 mM NaCl pH 7.4). Beads were boiled for 5 min in 60 μL of 1× Laemmli buffer to disrupt the bead-protein complex and elute proteins. Proteins were analyzed using Western blotting. 

### 4.7. Confocal Microscopy, Co-Localization, and Immunofluorescence

Transfected cells were fixed with 4% paraformaldehyde for 20 min at RT, washed with PBS, and mounted with ProLong Antifade Mountant (Thermo Fisher Scientific, Rockford, IL, USA) for imaging. Samples were visualized using a Zeiss LSM 700 confocal microscope using a Plan-Apochromat 63x/1.4 Oil DIC M27 objective. Zeiss ZEN 2010 program was used to control imaging specifications. The gap junction area was determined using ImageJ by tracing the gap junction area with a free hand tool followed by quantification using the measure tool. ImageJ software was also used to analyze co-localization data.

In the case of immunofluorescence, transfected cells were fixed with ice-cold 100% methanol for 10 min at RT. Cells were blocked using PBS supplemented with 2% BSA for 1 h at RT. Primary antibody, rabbit anti-HIS (Bethyl Laboratories Inc., Montgomery, TX, USA) at 1:1000 concentration, was diluted in PBS with 0.1% BSA and applied to cells for 1 h at RT. Cells were then incubated in the secondary antibody solution containing 2 μg/mL of Alexa Fluor 568 goat anti-mouse (Thermo Fisher Scientific, Rockford, IL, USA) and 2 μg/mL of Alexa Fluor 488 donkey anti-rabbit (Thermo Fisher Scientific, Rockford, IL, USA) in PBS with 0.1% BSA for 1 h at RT. Cells were washed in PBS and mounted with FluoroshieldTm (Sigma-Aldrich Chemie GmbH, Munich, Germany).

### 4.8. Förster Resonance Energy Transfer Analysis (FRET)

Neuro 2a cells were transiently transfected with Cx36-DsRed and Cx36-ECFP pair, Cx36-ECFP, and Cav-1-DsRed pair or DsRed and eCFP pair. Cells were fixed with 4% paraformaldehyde and mounted on coverslips. Zeiss LSM 700 confocal microscope was used under a previously established acceptor bleach protocol [71]. Baseline readings were recorded prior to the acceptor bleach protocol. DsRed tagged proteins were bleached using the 555 nm laser line (set to 100% intensity), and the resulting intensity change of CFP tagged proteins was measured using the 405 nm laser line. The experiment was conducted until the acceptor channel reached 10% of the initial intensity. FRET efficiency was calculated using the following FRET efficiency formula:

FRET*_eff_* = (*Dpost* −* Dpre*)/*Dpost* (1)

where *Dpost* is the average intensity after the bleach, and *Dpre* is the average intensity before the bleach. The threshold value of 10 nm distance was converted into FRET efficiency and was calculated to be 1.7% for DsRed and eCFP pair based on the reference distance between the two fluorescent tags (5.1 nm) [72].

### 4.9. Fluorescence Recovery After Photobleaching (FRAP)

Neuro 2a cells were seeded on 35 mM glass-bottom dishes and transfected and transfected with Cx36-EGFP and HA or Cx36-EGFP and Cav-1-HA. Live-cell imaging was performed at 37 °C in a live-cell imaging chamber, using a Zeiss 700 confocal microscope. Cx36-EGFP expressing cell pairs containing gap junctions were selected, and a time-lapse baseline image was recorded. The gap junction plaques were selected and bleached using the 488 nm laser line with the intensity set to 100% laser power. Images were taken every 1 s for 55 s post bleaching. The fluorescence recovery was calculated using the formula:

*F* = (*Ft* − *F*0)/(*Fi* − *F*0) (2)

where *F* is the normalized fluorescence at a given time point, *Ft* is the fluorescence intensity at t seconds, *Fi* is the fluorescence intensity immediately before bleaching, and *F*0 is the fluorescence intensity upon bleaching.

### 4.10. Total Internal Reflection Fluorescence (TIRF)

Neuro 2a cells were seeded on 35 mM glass-bottom dishes and transfected and transfected with Cx36-EGFP and HA or Cx36-EGFP and Cav-1-HA. A Zeiss Observer Z1 spinning-disk microscope with Zeiss 100X (Plan-Apochromat, DIC, M27, 1.46) oil immersion lens and Photometrics Evolve™512 camera, was used to perform time-lapse TIRF microscopy. Zen 2 (2014) software was used to control imaging specifications under a previously established protocol [14]. A live cell incubation chamber was used to maintain the temperature at 37 °C and CO2 levels at 5%. Images were acquired at a 512 × 512 pixel resolution in 1-s intervals for 60 s. Imaris (Zurich, Switzerland) program was used to track and analyze single particles expressing EGFP.

### 4.11. Statistical Analysis

Statistical analysis and data presentation were performed using GraphPad Prism 8. Values reported consist of mean ± SEM. Results shown derive from experimental replicates with *n* ≥ 3. Data were analyzed using the Wilcoxon–Mann–Whitney test.
